# Multi-Channel RF Supervision Module for Thermal Magnetic Resonance Based Cancer Therapy

**DOI:** 10.3390/cancers13051001

**Published:** 2021-02-28

**Authors:** Haopeng Han, Eva Oberacker, Andre Kuehne, Shuailin Wang, Thomas Wilhelm Eigentler, Eckhard Grass, Thoralf Niendorf

**Affiliations:** 1 Berlin Ultrahigh Field Facility (B.U.F.F.), Max Delbrück Center for Molecular Medicine in the Helmholtz Association (MDC), 13125 Berlin, Germany; haopeng.han@mdc-berlin.de (H.H.); eva.oberacker@charite.de (E.O.); thomas.eigentler@mdc-berlin.de (T.W.E.); 2 Humboldt-Universität zu Berlin, Institute of Computer Science, 10099 Berlin, Germany; grass@informatik.hu-berlin.de; 3 Department of Radiation Oncology and Radiotherapy, Charité Universitätsmedizin Berlin, 13353 Berlin, Germany; 4 MRI.TOOLS GmbH, 13125 Berlin, Germany; kuehne@mritools.de; 5 Beijing Deepvision Technology Co., Ltd., Beijing 100085, China; wangshuailin@deepvision-tech.com; 6 Technische Universität Berlin, Chair of Medical Engineering, 10623 Berlin, Germany; 7 IHP–Leibniz-Institut für Innovative Mikroelektronik, 15236 Frankfurt (Oder), Germany; 8 Experimental and Clinical Research Center (ECRC), A Joint Cooperation between the Charité Medical Faculty and the Max Delbrück Center for Molecular Medicine, 13125 Berlin, Germany

**Keywords:** glioblastoma multiforme, thermal magnetic resonance, hyperthermia, radio frequency heating, power meter, phase meter, hyperthermia treatment planning, patient displacement

## Abstract

**Simple Summary:**

Glioblastoma multiforme (GBM) is the most lethal brain tumor. Combining hyperthermia with chemotherapy and/or radiotherapy improves survival of GBM patients. For radio frequency (RF)-induced hyperthermia, the RF signals’ power and phase need to be supervised to achieve a precise formation of the power deposition focal point, accurate thermal dose control, and safety management. Patient position during treatment also needs to be monitored to ensure the efficiency of the treatment and to avoid adverse effects in healthy tissue. This work demonstrates the development, implementation, evaluation, validation, and application of a multi-channel RF supervision module that meets the technical requirements of hyperthermia and provides a cost-effective solution for broad-band RF signal supervision and patient monitoring. It is a key component for a hyperthermia hardware system and facilitates future thermal magnetic resonance applications that integrate RF-induced heating, in vivo temperature mapping, and anatomic and functional imaging in a single RF applicator.

**Abstract:**

Glioblastoma multiforme (GBM) is the most lethal and common brain tumor. Combining hyperthermia with chemotherapy and/or radiotherapy improves the survival of GBM patients. Thermal magnetic resonance (ThermalMR) is a hyperthermia variant that exploits radio frequency (RF)-induced heating to examine the role of temperature in biological systems and disease. The RF signals’ power and phase need to be supervised to manage the formation of the energy focal point, accurate thermal dose control, and safety. Patient position during treatment also needs to be monitored to ensure the efficacy of the treatment and avoid damages to healthy tissue. This work reports on a multi-channel RF signal supervision module that is capable of monitoring and regulating RF signals and detecting patient motion. System characterization was performed for a broad range of frequencies. Monte-Carlo simulations were performed to examine the impact of power and phase errors on hyperthermia performance. The supervision module’s utility was demonstrated in characterizing RF power amplifiers and being a key part of a feedback control loop regulating RF signals in heating experiments. Electromagnetic field simulations were conducted to calculate the impact of patient displacement during treatment. The supervision module was experimentally tested for detecting patient motion to a submillimeter level. To conclude, this work presents a cost-effective RF supervision module that is a key component for a hyperthermia hardware system and forms a technological basis for future ThermalMR applications.

## 1. Introduction

Glioblastoma multiforme (GBM) is the most common malignant primary brain tumor, accounting for 14.6% of all primary brain tumors and 57.3% of all gliomas [1]. Less than 5.8% of patients survive five years post diagnosis, which renders GBM the most lethal type of brain tumor [1]. Surgical resection followed by radiotherapy and chemotherapy remains the mainstay of care for GBM patients [2,3]. Adding thermal therapy to the standard treatment of GBM could improve prognosis [4]. Mild regional hyperthermia (HT; 40–44 °C for 60–90 min) is a clinically proven adjuvant anti-cancer treatment in conjunction with radiotherapy and/or chemotherapy that significantly improves survival [5,6,7,8,9]. Non-invasive HT modalities targeting the GBM mainly include magnetic nanoparticle hyperthermia (MNH), focused ultrasound-based hyperthermia, and radio frequency (RF)-induced hyperthermia. A significant improvement of survival was reported for GBM patients receiving chemotherapy together with RF-induced HT [10]. Thermal magnetic resonance (ThermalMR) is an HT variant that integrates RF-induced heating [11,12,13,14,15], in vivo temperature mapping using MR thermometry (MRT) [16,17,18,19], anatomic and functional MR imaging (MRI), and the option for x-nuclei MRI in a single, multi-purpose RF applicator that permits supervised targeted temperature modulation.

Targeted RF-induced heating is based on electromagnetic waves transmitted with a multi-channel RF applicator that are sought to constructively interfere in the targeted heating volume while keeping RF power deposition outside of the target to a minimum to preserve healthy tissue. The interference pattern is governed by the frequency, power, and phase settings of the RF applicator. To manage RF power deposition in the target volume, safety, and thermal dose control, the RF signals’ power and phase need to be supervised in real-time. Commercial or in-house developed supervision devices are used to monitor RF signals in hyperthermia or in MRI [20,21,22,23]. These supervision devices lack the ability to measure the phase of RF signals and/or typically operate at a single frequency. The use of multiple distinct or a broad range of frequencies improves the quality of RF heating [24,25,26,27,28,29]. To ensure precise thermal dose control in multi-frequency applications, it is essential to assess and compensate for the frequency-dependent characteristics of RF meters.

Quality assurance is of profound importance for HT delivery. Factors that could potentially impede the quality of RF-induced hyperthermia include unbalanced RF power amplifiers, antenna mismatches, inaccuracies in cable lengths, antenna location offsets, and phase errors in the RF chain. These factors usually cannot be modeled during treatment planning but can be compensated with calibration algorithms or corrected using a feedback control loop [30,31]. These approaches require the accurate measurement of the power level and phase of the RF signals connected to the RF applicator. For this reason, the monitoring of the forward and reflected RF signals of state-of-the-art high-density RF applicators asks for multi-channel RF supervision modules. 

Patient motion has the potential to severely impede the quality of HT delivery, with bulk head motion being detrimental for the hyperthermia treatment planning (HTP) of glioblastoma multiforme. For HTP, the position of the patient model is assumed to accurately reflect the position during the clinical HT delivery [32,33]. Patient displacement from the optimized setting might impair HT quality and may even constitute a potential safety hazard. Consequently, patient motion should be monitored during HT delivery to ensure the efficiency and safety of hyperthermia treatments. For HT delivery, RF antennas are strongly coupled to the underlying tissue. The antennas’ sensitivity to changes in the conductivity/impedance of the underlying structure within their electric fields facilitates motion detection by measuring the returned signals from the RF applicator [34,35]. Increasing the number of RF channels enhances HT quality and the degrees of freedom for motion tracking using a multi-channel RF supervision module.

To summarize, advancing high-fidelity HT delivery requires pioneering strategies that exploit high-density, multi-channel RF antenna arrays with independent frequency, amplitude, and phase control for each channel; a wider range of RF frequencies; and motion detection/correction approaches. Recognizing these opportunities and challenges, this work reports on the design, implementation, evaluation, validation, and application of a multi-channel RF supervision module tailored for real-time RF power and phase monitoring and regulation. This setup was designed to operate in a frequency range from 10 MHz to 2.7 GHz with phase and power resolutions of 7.2 × 10^−3^ (°) and 2.6 × 10^−3^ dBm, respectively, and it could be used as an RF supervision module for ThermalMR. To examine the RF supervision module’s applicability to RF heating, experiments were conducted in a phantom setup with and without the supervision module regulating the RF signals. Monte-Carlo simulations were performed to examine the impact of power and phase errors on hyperthermia performance. To detail the possible impact of bulk head motion on HT delivery in the head, electromagnetic field (EMF) simulations were performed using (i) a human voxel model that was modified to include an intracranial sphere mimicking a small tumor in the right parietal region of the brain and (ii) a human voxel model that was generated from a computed tomography scan of a GBM patient. Following the conclusions obtained from the EMF simulations of the human head voxel models, the RF supervision module’s applicability for motion detection was demonstrated with an experimental head model. 

## 2. Materials and Methods

### 2.1. System Design

**Figure 1:**
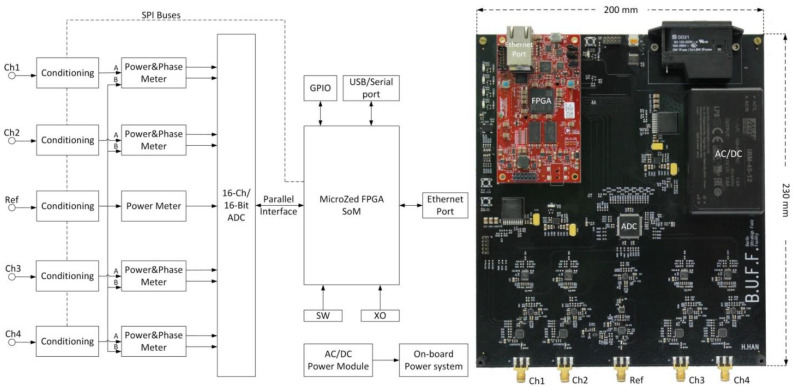
Left: System block diagram of the supervision module. One power meter was implemented in the reference channel whereas identical power and phase meters were implemented in the radio frequency (RF) input channels. Two serial peripheral interface (SPI) buses were routed from the FPGA (field-programmable gate array) to the conditioning chips for configuration. The FPGA receives data from the analog-to-digital converter chip through its parallel interface. The Ethernet interface was utilized for data exchange. The on-board power system distributes various DC (direct current) power supplies to components on the board. GPIO: general-purpose input/output; SoM: system-on-module; SW: switches; XO: crystal oscillator; ADC: analog-to-digital converter. Right: A photo of the supervision module. The red module is a system-on-module unit (AES-Z7MB-7Z020-SOM-I-G, Avnet, Phoenix, AZ, USA). Please note that the photo is rotated by 90 degrees versus the block diagram shown on the left-hand side.

The supervision module contains four RF input channels and one reference signal input channel and supports a frequency range from 10 MHz to 2.7 GHz. Figure 1 shows the block diagram and the implemented hardware. The four RF input channels were identical in circuit design, with the impedance matched to 50 Ohm. There was no difference in the signal propagation delay between the four RF input signals. The signal conditioning circuits consisted of attenuators in the RF input channels or a voltage-controlled amplifier (VGA) in the reference channel. Each RF input signal was firstly fed to a wideband digital step attenuator (F1956, 1 MHz to 4 GHz, Renesas Electronics, Tokyo, Japan). The reference signal input was firstly connected to a VGA chip (ADL5330, 10 MHz to 3 GHz, Analog Devices, Norwood, MA, USA). The gain of the VGA is linearly adjustable in decibels and controlled by the voltage output of a 16-bit digital-to-analog converter (AD5683, Analog Devices). The reference signal was conditioned to fit the range in which the RF power and phase meter chips operated with the highest linearity. After conditioning, the reference signal was split into five length- and impedance-matched routes; four fed to input B of four power and phase meter chips (AD8302, 0–2.7 GHz, Analog Devices) and one connected to a logarithmic power meter (ADL5513, 1 MHz to 4 GHz, Analog Devices). Each RF input signal was routed to input A of an AD8302 chip. The meter chip AD8302 measures the power and phase differences between RF signals at its input A and B. The measured power level and phase information is contained in the voltage level of the meter’s outputs. All analog outputs from the meters were fed to a 16-channel, 16-bit analog-to-digital converter (ADC; AD7616, Analog Devices). After digitization, the signals were sent to a field-programmable gate array chip (FPGA; XC7Z020, Xilinx, San Jose, CA, USA), which manages the whole system and is the core of a system-on-module unit (AES-Z7MB-7Z020-SOM-I-G, Avnet, Phoenix, AZ, USA). Customized FPGA logic utilizing direct memory access (DMA) was developed to interface the ADC chip. Digital low-pass filters were implemented with the FPGA logic to filter the signals. AXI (Advanced Extensible Interface) bus-based IP (intellectual property) cores were developed using the programmable logic resources in the FPGA to configure the signal conditioning circuits.

**Figure 2:**
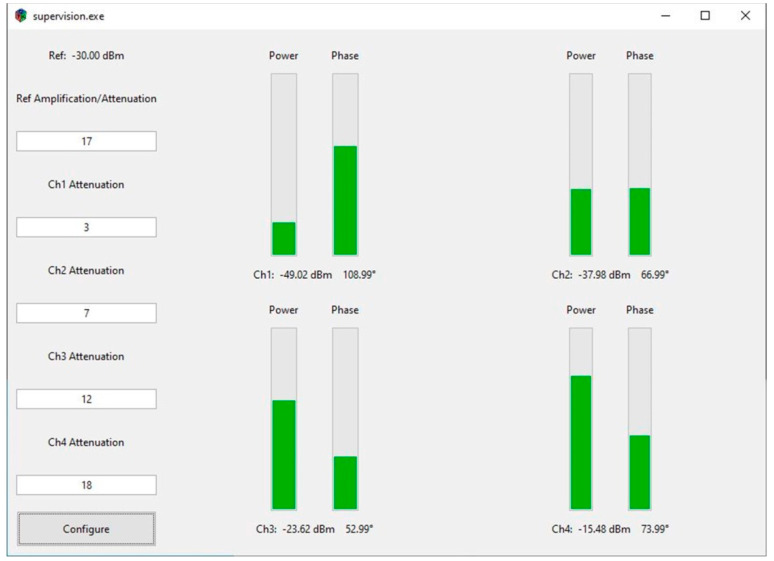
Graphical user interface (GUI). Users can configure the conditioning chips on the left side of the GUI. The RF power and phase information are displayed on the right of the GUI. Progress bars are used to show the relative relationship between channels.

The module runs bare metal embedded software developed in C on the dual-core ARM processor inside the FPGA chip. A UDP (User Datagram Protocol) server was implemented for data exchange. The power and phase information of all channels was encapsulated in one UDP packet. A graphical user interface (GUI; Figure 2) that runs on the host computer was developed in Haskell to monitor the measured RF power and phase information, as well as to configure the signal conditioning settings including attenuation and amplification. The GUI communicates with the UDP server in the supervision module through an Ethernet connection.

### 2.2. System Characterization

For the evaluation of the multi-channel RF supervision module, all tests were conducted at room temperature (22 °C) with the RF supervision module warmed up for 30 min. Coaxial cables (length = 1 m; 135101-01-M1.00, Amphenol, Wallingford, CT, USA) were used to connect the signal generators and the supervision module. All the calibrations were conducted for 91 frequency points ranging from 100 to 1000 MHz in increments of 10 MHz. For the calibration of the logarithmic power meter chip ADL5513, a calibrated power signal generator (SMGL, R&S, Munich, Germany) was connected to the reference channel, with the signal conditioning circuits bypassed. Sinusoid signals with power levels in the range from −70 to 10 dBm and a power increment of 1 dBm were generated to feed the meter chip. In total, 81 readings from the power meter were recorded for each frequency. The phase meter AD8302 was calibrated with a calibrated 4-channel arbitrary waveform generator (AWG; M8190A, Keysight, Santa Rosa, CA, USA). Channel 1 of the AWG generated a sinusoid signal with a power level of −30 dBm at input B of the chip as the reference signal. Channel 2 of the AWG generated a coherent sinusoid signal that was connected to input A of the meter chip under calibration. The phase of channel 2 relative to channel 1 was varied in the range of 0°–359° using a 1° increment, and 360 readings from the phase meter were recorded for each frequency. For calibrating the power meter function of the AD8302 chip, a −30 dBm sinusoid signal generated by the AWG was connected to input B of the chip. Sinusoid signals generated by the SMGL power signal generator with power levels in the range from −70 to 10 dBm and a step size of 1 dBm were connected to input A of the meter chip. The AWG and the power signal generator shared the same external reference clock source (CDA-2990, National Instruments, Austin, TX, USA). 81 readings from the power meter were recorded for each frequency. All the recorded data were fed into MATLAB R2017b (The MathWorks, Natick, MA, United States) for curve fitting based on the least-squares method. The fitting results for the two power meters were verified with measurements obtained with a power sensor (NRP18T, R&S, Munich, Germany).

An application was developed in C to test the throughput of the UDP server implemented in the supervision module. The application runs on a desktop computer (Intel Core i7-7700, 16 GB RAM, Gigabit Ethernet) and constantly requests power and phase readings from the supervision module. The number of UDP packets (each packet contains information for all channels) was counted for ten minutes to get an average sampling rate of the supervision module.

### 2.3. RFPA Characterization and RF Heating Experiments

**Figure 3:**
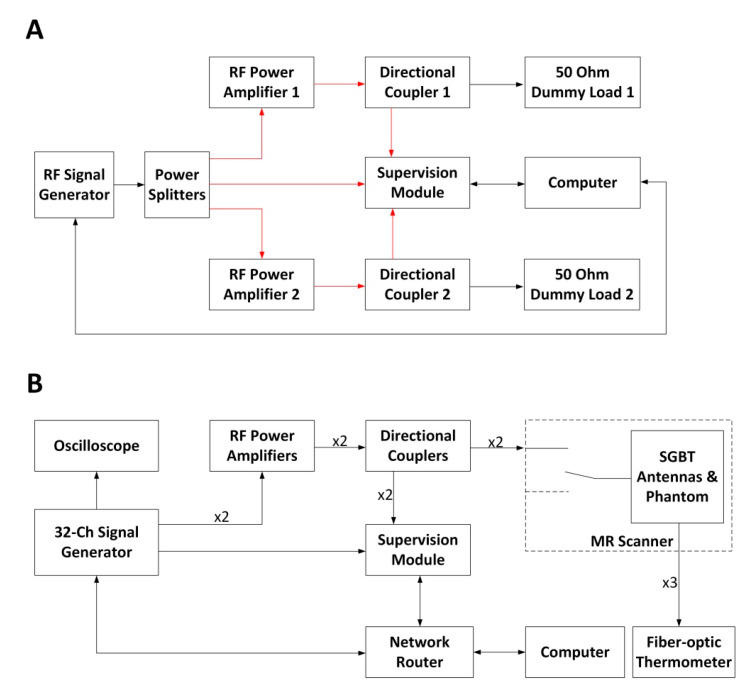
(**A**) Schematic of the experimental setup used for the RFPA (RF power amplifier) characterization. The connections illustrated in red share the same cable length. The power splitters and the directional couplers were characterized using a vector network analyzer (ZVT8, R&S) so that the unbalances in the two RF signal chains could be compensated during the characterization of the RFPAs. The computer controls the signal generator via a GPIB (general purpose interface bus) interface. (**B**) Schematic of the experimental setup in the RF heating experiments. Four coherent RF signals at 400 MHz were generated with a custom-built RF signal generator. One signal was connected to an oscilloscope for monitoring. Two signals were connected to two RFPAs, and the last one was connected to the supervision module as the reference signal. The supervision module monitored and regulated the forward coupled outputs of the directional couplers. The two RF signal chains (from the signal generator to the self-grounded bow-tie (SGBT) antennas) shared the same electrical length. MR: magnetic resonance.

The supervision module was utilized to characterize and supervise two home-built RF power amplifiers (RFPAs) that were used for RF heating. The two RFPAs shared the same design that had a bandwidth of 400 MHz (100–500 MHz) and a nominal gain of 50 dB. For the characterization of the amplifiers, a calibrated RF signal generator (SMGL, R&S) generated a sinusoid signal that was split equally with power splitters (ZFSC-2-1W-S+, Brooklyn, NY, USA). The split RF signals were used as input for the RF amplifiers and as the reference signal for the supervision module (Figure 3A). The gain and phase of the RFPAs were characterized versus the frequency and amplitude of the input RF signals. For testing the RFPAs’ performance against frequency, two frequency sweeps with different input power levels (0 and −10 dBm) in the range of 100–500 MHz and a step size of 1 MHz were conducted. For testing the RFPAs’ performance against input amplitude, two amplitude sweeps with different signal frequencies (300 and 400 MHz) in the range from −30 to 0 dBm and a step size of 0.5 dBm were conducted. The power levels and phases of the forward coupled outputs of the directional couplers were recorded with the supervision module. The unbalances in the two RF paths introduced by the power splitters and the directional couplers were compensated in post-processing. 

For RF heating, two experiments were conducted with and without the supervision module regulating the RF signals (f = 400 MHz). The schematic of the experimental setup is illustrated in Figure 3B. Two coherent RF signals generated from the custom-built RF signal generator [15] were fed to the same two RFPAs that were characterized with the supervision module. The amplified signals were connected to two wideband self-grounded bow-tie (SGBT) antennas [14] through two directional couplers (BDC0810-50/1500, BONN Elektronik, Holzkirchen, Germany) and the feed-through penetration panel of the MR scanner room. The antennas were positioned opposite to each other and applied to a muscle-mimicking agarose phantom (length = 160 mm, width = 116 mm, height = 178 mm; ε_r_ = 54.32, and σ = 0.25 S/m [15]). The phantom was placed in the isocenter of a 7.0 T human MR scanner (Magnetom, Siemens Healthineers, Erlangen, Germany).

The first RF heating experiment was conducted with the supervision module being in the control loop regulating the RF signals. The set-point for the RF power level at the feeding port of the SGBT antenna was set to 42.5 dBm (17.78 W) for each RF channel. The two RF channels were regulated to have the same phase at the feeding ports of the antennas. For the second heating experiment, the supervision module was not included in the control loop. The signal generator generated two RF signals with the same phase. The power level at the feeding port of the SGBT antenna was set to 42.5 dBm (17.78 W) for each RF channel at the beginning of the experiment. The RF power was applied to the phantom for 10 min for both RF heating experiments. MR thermometry utilizing the proton-resonance-frequency-shift (PRFS) approach [36,37] (TR = 99 ms, TE1 = 2.73 ms, TE2 = 6.71 ms, and voxel size = 1.0 × 1.0 × 5.0 mm^3^ [15]) at 297.2 MHz was conducted before and after each experiment. Vegetable oil was used as a reference to correct the magnetic field drifts [15,38]. Fiber optic temperature sensors (Neoptix, Quebec, QC, Canada) were used to validate the MRT results [15]. For benchmarking experimental data with numerical simulations, temperature simulations using the same frequency, power, and phase settings (400 MHz, 42.5 dBm, and ϕ = 0° for both channels at the feeding port of the SGBT antenna) were performed in CST Microwave Studio 2018 (Computer Simulation Technology GmbH, Darmstadt, Germany). For this purpose, the phantom configuration used in the heating experiments was incorporated into the numerical simulations. The CST thermal transient solver was adopted with a hexahedral mesh type. The maximum mesh size was set to 2.0 × 2.0 × 2.0 mm^3^, which was sufficient for the problem since further decreasing the maximum mesh size by 10% did not yield substantial changes (<0.01%) in the simulation results [15]. An open boundary condition was used with the ambient temperature 20 °C set at the boundary as constant temperature.

### 2.4. Head Motion Detection

**Figure 4:**
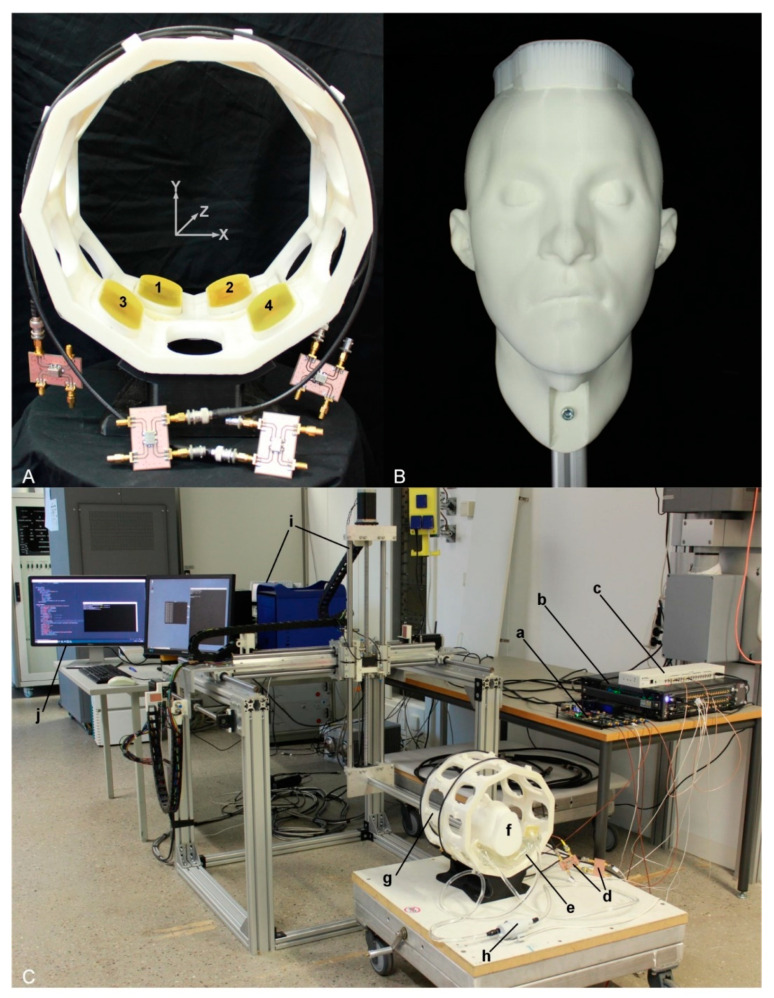
Experimental setup used for head motion detection. (**A**) Annular antenna holder in a two-ring arrangement (inner diameter = 24 cm) including ten antenna positions for each ring. Four SGBT antennas (two for each ring) were installed in the holder, as illustrated by the numbers 1–4. The antennas were connected to the signal generator via home-built directional couplers. (**B**) A 3D printed head model installed to an aluminum profile and used as an object under investigation. (**C**) The experimental setup, (**a**) supervision module, (**b**) custom-built RF signal generator, (**c**) clock distributor, (**d**) home-built directional couplers, (**e**) water pad, (**f**) head model, (**g**) antenna holder, (**h**) water pump, (**i**) the COSI Measure setup [42], and (**j**) computer for interacting with the equipment.

To detail the possible impact of patient movements on HT delivery, EMF simulations were performed (f = 297 ± 50 MHz) using Sim4Life V3.4 (ZurichMedTech, Zurich, Switzerland). For this purpose, the heads of two human voxel models were inserted into the middle of the annular phased array RF applicator (Figure 4A), with a cylindrical water bolus surrounding the models to fill the air gap between the antenna array and the head model. First, the human voxel model “Duke” from the virtual family [39] (IT’IS Foundation, Zürich, Switzerland) was used. This model was modified to include an intracranial sphere (diameter = 4 cm, ε_r_ = 66.5, and σ = 1.15 S/m [39]) mimicking a small tumor in the right parietal region of the brain (target volume = 33.5 mL). The second human voxel model was used to mimic a clinical scenario. This model was generated from a computed tomography scan of a patient with glioblastoma multiforme [40]; it encompassed a large clinically relevant target volume (500 mL). Hereafter, this model is referred as the clinically relevant model (CRM). The initial positioning of the head models in the RF applicator was chosen so that the center of the target volume aligned with the center of the applicator in head-feet-direction (Z). The resolution of the simulations was limited to a maximum step size of 3 mm within the skull. A much finer resolution of down to less than 0.5 mm was applied to resolve the bent and triangular shape of the SGBT antennas.

After the simulation, E- and H-field data were exported channel-wise and isotropically rebinned to a resolution of 3 mm^3^, and SAR_10g_ (specific absorption rate) matrices were calculated [11,41]. For HTP, power and phase settings for each channel were optimized using the multiplexed vector field shaping (MVFS) method [29] to maximize RF power deposition in the target volume while limiting the exposure in healthy tissue. The targeted SAR in the tumor was set to 100 W/kg, while the SAR limit in healthy tissue was set to 40 W/kg. To investigate the impact of patient malpositioning, a second set of simulations was performed after the longitudinal displacement of the head model (ΔZ = 5 mm). Phase and amplitude settings obtained for the originally targeted position were applied to these new simulation results.

The obtained RF power deposition regarding both the upkeep of the therapeutic exposure and the sparing of the healthy tissue was assessed. The maximum local SAR_10g_ was evaluated in the tumor and the healthy tissue, together with the respective mean SAR_10g_ value. The volume power density P_tumor_/V_tumor_, being the quantity which governs the actual heating of the tissue, assesses the overall RF power deposition in the target volume. Normalization with the tumor volume makes it comparable across various models. The SAR amplification factor (SAF; [24]) quantifies the overall sparing of the healthy tissue by relating the mean exposure in the target volume to the mean exposure of the healthy tissue (SAF = SAR_10g,mean_(tumor)/SAR_10g,mean_(healthy)). The tumor-to-hotspot quotient (THQ; [43]) considers local RF maxima in the healthy tissue to be the critical factor rather than overall exposure and relates the mean exposure in the target volume to the first volume percentile of local SAR_10g_ values (THQ = SAR_10g,mean_(tumor)/P1 (SAR_10g_(healthy))). TC (SAR_tumor_ > SAR_Lim_) measures the target coverage with a local RF exposure greater than what is allowed in the healthy tissue and assesses the distribution of the RF power deposition in the target volume. This is of particular importance in large target volumes, where local maxima could form while other regions could exhibit greater underexposure than expected by the local or averaged SAR values. 

Following the conclusions obtained from the EMF simulations of the human head models, the RF supervision module’s applicability for motion detection was examined in experiments. For this purpose, a 3D printed human head model was used as an OUI (object under investigation). The OUI was fixed to an open source 3D multipurpose measurement system (COSI Measure) [42] through an aluminum profile (Figure 4B). The human head model was placed onto the SGBT antennas with a pad of water placed in between. The water in the pad was circulated with a pump (1005.02.00, Comet, Paynesville, MN, USA) to mimic a clinical HT treatment setup. The antennas were installed in an annular holder (Figure 4A). The signal generator generated five RF signals at 297 MHz with a power level of 0 dBm. Four signals were fed to the four antennas via home-built directional couplers; one was fed to the supervision module as a reference signal. To examine the RF supervision module’s applicability for tracking, three movements with displacements of 0.5, 1.0, and 5.0 mm were performed along the X-, Y- and Z-axes using COSI Measure. The four reflected signals were measured and recorded with the supervision module.

### 2.5. Analysis of Impact of Power and Phase Errors on Hyperthermia Performance

To analyze the impact of power and phase errors of the RF signals on the performance of a hyperthermia system, Monte-Carlo analyses were conducted on the power and phase settings obtained from the EMF simulations and optimizations targeting the clinically relevant model introduced in Section 2.4. The optimum solution was perturbed with excitation error vectors of different magnitudes and phases. Ultimately, 256 Monte-Carlo simulations over an error vector space of 0–15% power error (per channel) and 0–15° phase error (per channel) were analyzed, with each run containing 1 million randomized vectors. The focusing efficiency was calculated by dividing the peak SAR_10g_ in the tumor by the peak SAR_10g_ in healthy tissue. At the optimum solution, the focusing efficiency was ~2.1, i.e., peak SAR_10g_ in the tumor was about twice as high as the peak SAR_10g_ in healthy tissues. Any error in the system would both potentially increase healthy SAR_10g_ and decrease tumor SAR_10g_, thus degrading this metric below 2.1. Based on this metric, a statistical analysis of how well a system with a given error range performs compared to a perfect system was carried out. 

## 3. Results

### 3.1. System Characterization

**Figure 5:**
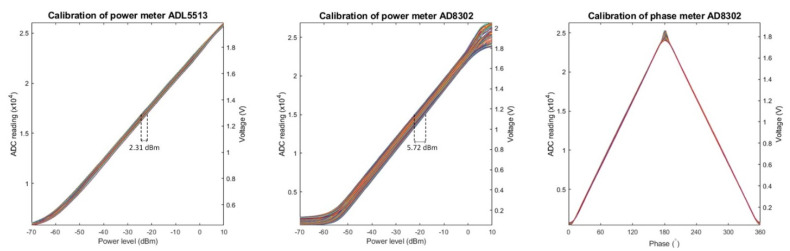
Plots of the measured data obtained during the characterization of the RF supervision module. Each graph includes 91 curves each representing the results obtained for one tested frequency. Left: The ADC readings for the power meter ADL5513 were plotted against corresponding power levels of the tested signals. For the same ADC reading, a maximum variation of 2.31 dBm was observed among the curves. Middle: The ADC readings for the power meter AD8302 were plotted against corresponding power levels of the RF signals. For the same ADC reading, a maximum variation of 5.72 dBm was observed for the tested discrete frequencies. Right: The ADC readings for the phase meter AD8302 were plotted against corresponding phases of the tested signal. The curves are symmetrical around a phase of 180°.

Figure 5 illustrates the calibration results obtained for system characterization. For the power meter in the reference channel (ADL5513), an excellent linear relationship (R^2^ = 1.00) between the ADC readings and the power levels (range: from −50 to 0 dBm) was observed for all 91 tested frequency points. The signal frequency had an impact on the results. A maximum variation of 2.31 dBm was observed for the same ADC reading among the tested frequency points. Equation (1) describes curve fitting results of the meter (ADC_ref_ represents the reading from the ADC and Freq is the signal frequency in MHz). A theoretical resolution of 3.6 × 10^−3^ dBm was obtained for this meter. A mean absolute error of 0.01 mW with a standard deviation of 0.02 mW was calculated using Equation (1) against the data measured with the commercial power sensor (NRP18T, R&S, Munich, Germany).

(1) $$PowerLevel_{ref}=\left(3.6\times ADC_{ref}+1.427\times Freq-86599\right)\times 10^{-3}\left(dBm\right)$$

For the power meter in RF input channels (AD8302), an excellent linear relationship (R^2^ = 1.00) between the ADC readings and the power levels (from −50 to 0 dBm ) was also found. The curves were scattered depending on the frequency of the input signals. A maximum variation of 5.72 dBm was observed for the same ADC reading among the tested frequency points. Equation (2) describes the curve fitting results of this power meter (ADC_RF_input_ represents the reading from the ADC and Freq is the signal frequency in MHz). The theoretical resolution of this meter is 2.6 × 10^−3^ dBm. A mean absolute error of 0.02 mW with a standard deviation of 0.04 mW was calculated using Equation (2). The mean absolute tolerance calculated against the data measured with the commercial power sensor (NRP18T) was 12%.

(2) $$PowerLevel_{RF_{input}}=(2.6\;\times ADC_{RF_{input}}+8.337\;\times 10^{-5}\times Freq^{3}-0.135\times Freq^{2}+\;57.175\times \;\mathrm{Freq}-\;64623)\;\times 10^{-3}\;\left(dBm\right)$$

For the phase meter in RF input channels (AD8302), a linear relationship (R^2^ = 1.00) between the ADC readings and the phases was observed in the range of 5°–175°, with almost no influence from the frequency of the signals. The phase curves were symmetrical around a phase of 180°. This complied with the characteristics of the meter chip. Equation (3) describes the calibration results of the phase meter (ADC_phase_ represents the reading from the ADC). The theoretical resolution of this meter is 7.2 × 10^−3^ (°). A mean absolute error of 0.50° with a standard deviation of 0.41° was calculated using Equation (3) against the measured data.

(3) $$Phase=\left(7.2\times ADC_{phase}+2648\right)\times 10^{-3}\left({}^{\circ}\right)$$

The maximum throughput of the UDP server was 28.4 packets per second, with the power and phase information of all channels contained in one packet.

### 3.2. RFPA Characterization and RF Heating Experiments

**Figure 6:**
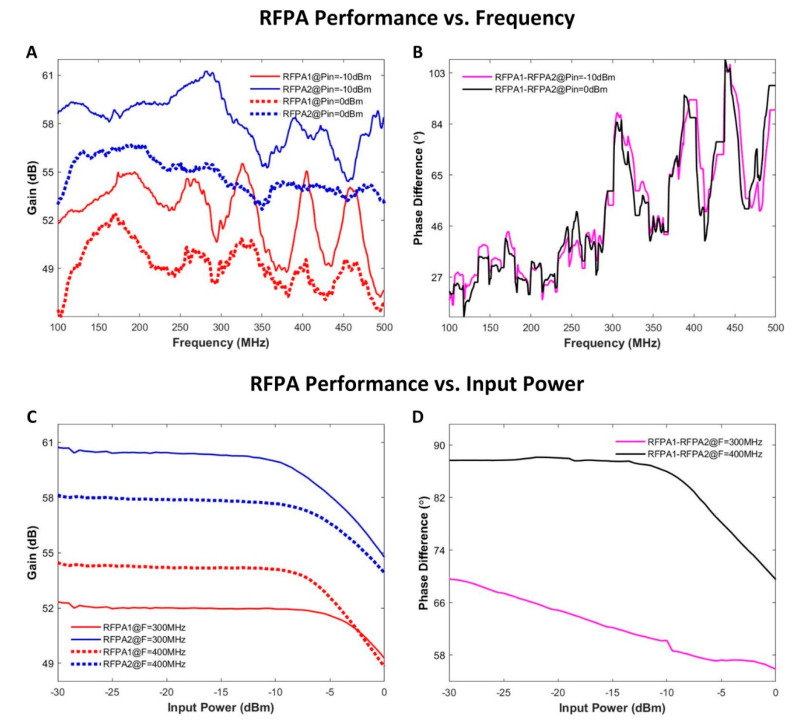
Summary of RFPA characterization results. (**A**) The gain of the two RFPAs was measured at two input levels (−10 and 0 dBm) for a frequency range of 100–500 MHz in increments of 1 MHz. (**B**) The phase difference between the outputs of the two RFPAs against frequency at different input power levels. (**C**) The gain of the two RFPAs was measured at two frequencies (300 and 400 MHz) with the input power level varying from −30 to 0 dBm using an increment of 0.5 dBm. (**D**) The phase difference between the outputs of the two RFPAs against input power level at different frequencies.

The results of the characterization of the RFPAs using the supervision module are displayed in Figure 6. The two RFPAs (identical in design and implementation according to the vendor) demonstrated different behaviors for the variation of the signal frequency and the variation of the input power level (Figure 6A). RFPA 2 yielded an average gain that was 5.5 dB superior to RFPA 1 for both input signal levels across the tested frequency range. The maximum variation of the gain of RFPA 1 along frequency was 8.3 dB. For RFPA 2, a maximum variation of the gain of 6.9 dB was observed. With the input signals set to identical phases for each RFPA, large phase differences were found between the output signals of the RFPAs (Figure 6B). These phase differences changed with frequency. When the frequency of the input signals was fixed and the amplitudes were altered from −30 to 0 dBm, the two RFPAs showed different gains (Figure 6C). The gain difference was as large as 8.4 dB for signals at 300 MHz. For an RF input signal at 400 MHz, RFPA 1 provided a higher gain compared to 300 MHz. However, RFPA 2 behaved differently in this regard. At 400 MHz, the phase difference between the outputs of the two RFPAs maintained roughly constant (~88°) in the linear range of the RFPAs (Figure 6D). The phase difference between the outputs of the two RFPAs showed a linear decrease when sweeping the RF input power level at 300 MHz.

**Figure 7:**
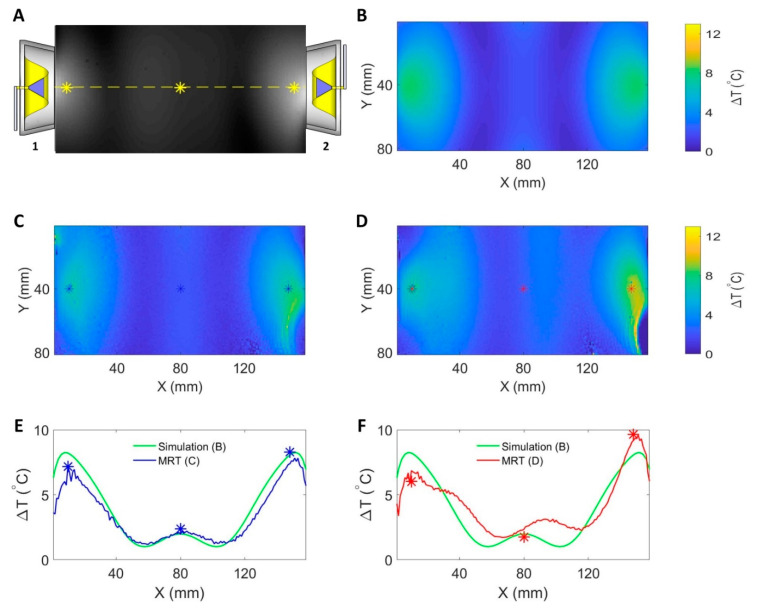
Summary of RF induced temperature changes (ΔT) obtained from numerical simulations and experiments (f = 400 MHz, t = 10 min, and P_in_ at port = 17.78 W) with each RFPA being connected to an SGBT antenna. (**A**) The position of the antennas and an axial MR image through the center of the phantom. The yellow line indicates the coronal centerline. The yellow stars mark the positions of the fiber optic temperature sensors. A transversal slice in the middle of the phantom aligned with the center of the RF applicator was selected for data analysis. (**B**) The temperature changes obtained from the temperature simulation. The middle row shows maps of temperature changes derived from MR thermometry for RF heating with (**C**) and without (**D**) the supervision module in the control loop regulating the RF signals. The stars in (**C**,**D**) indicate the positions of the fiber optic temperature sensors. The bottom row depicts ΔT profiles obtained for the centerline drawn through the center slice of the phantom for temperature simulations (**B**), experimental RF heating with (**C**) and without (**D**) the supervision module in the control loop. The blue and red stars indicate readings from the temperature sensors. A constructive interference pattern that was similar to the simulation result was observed in the middle of the phantom when the supervision module was activated in the loop (**E**). The interference pattern was distorted when the supervision module was not in the loop (**F**). For this case, the peak of the experimental interference pattern was shifted 16 mm to the right versus the reference obtained from the temperature simulation.

Figure 7 summarizes the results derived from numerical temperature simulation and experiments using the RF heating setup at 400 MHz. Figure 7B depicts the temperature changes obtained from the numerical temperature simulation. With both RF channels running the same settings at the ports of the SGBT antennas, a constructive interference pattern was generated in the middle of the phantom. RF heating experiments using the supervision module for the regulation of the RF signals showed a very good agreement between MR thermometry (Figure 7C) and the temperature simulation (Figure 7B). The temperature profile obtained along the centerline across the phantom (Figure 7E) demonstrated the same interference pattern created in the experiment compared with the interference pattern in the simulation. MRT detected a maximum temperature increase of ΔT = 2.21 °C in the middle of the phantom. The experimental interference pattern was distorted (Figure 7D) compared to the temperature simulation when the supervision module was not in the loop. For this case, the peak temperature rise in the middle of the phantom was ΔT = 3.14 °C. The peak of the experimental interference pattern was shifted 16 mm to the right (Figure 7F) versus the reference provided by the temperature simulation.

### 3.3. Head Motion Detection

**Figure 8:**
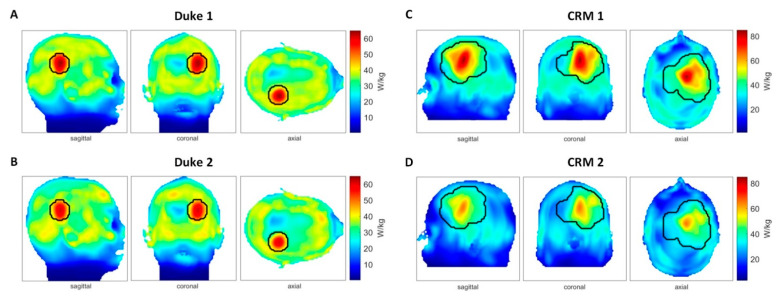
Impact of head displacement on RF heating obtained from electromagnetic field (EMF) simulations using human head models without (center position) and with movements (5 mm off-center position). (**A**) SAR_10g_ (specific absorption rate) maps (maximum intensity projection: MIP) obtained for the Duke model placed in the center of the RF applicator (Duke 1). The phase and amplitude setting of the RF heating was optimized for this position. (**B**) SAR_10g_ maps (MIP) derived from the Duke model being shifted 5 mm upwards (Duke 2). Phase and amplitude settings were identical with the used settings when the head model was placed in the center position. Several hotspots were generated in healthy tissue. (**C**) SAR_10g_ maps (MIP) obtained for the clinically relevant head model placed in the center of the RF applicator (clinically relevant model (CRM) 1). The phase and amplitude setting of the RF heating was optimized for this position. (**D**) SAR_10g_ maps (MIP) derived from the clinically relevant head model using an off-center (5 mm shift upwards) positioning (CRM 2) in conjunction with the same phase and amplitude settings used for (**C**).

To detail the impact of head movements on HT delivery, EMF simulations were performed at f = 297 ± 50 MHz. The resulting maximum intensity projections of the SAR_10g_ distributions of the RF heating with and without head displacement are outlined in Figure 8. Table 1 surveys the metrics used for the evaluation of the RF heating results with and without head displacement. Heating results obtained for 5 mm off-center positions (Duke 2 and CRM 2) showed a lower maximum SAR in the tumor compared with the centered models (Duke 1 and CRM 1). The CRM yielded a relative difference of ΔSAR_10g,max_ = 22.3% between the center and off-center position of the head. For SAR_10g,max_ in healthy tissue, the heating results with patient displacement (Duke 2 and CRM 2) exceeded the SAR limit set for the healthy tissue (SAR_lim_(healthy) = 40 W/kg). The heating results with the centered positions (Duke 1 and CRM 1) outperformed their off-center counterparts regarding SAR_10g,mean_. In healthy tissue, the SAR_10g,mean_ values obtained for the center and off-center position of the Duke model were similar. The simulation of the CRM revealed a higher SAR_10g,mean_ for the center position versus the off-center position. The SAF quantifies the ratio between the average RF power deposition in the tumor region versus healthy tissue. Larger values indicate that an RF power is more focused to the tumor area. TC(SAR_tumor_ > SAR_Lim_) is the proportion in the tumor volume that has a higher SAR than the limit set for the healthy tissue. It is also an indicator of the heating efficiency. For CRM 2, a drop of 33.45% of target coverage was observed. The overall RF power deposition in the target volume (P_tumor_/V_tumor_) decreased by 4.38% and 15.43% for the shifted Duke model and for the CRM, respectively. The THQ dropped by 9.84% and 8.18% for the shifted Duke model and for the CRM, respectively.

**Figure 9:**
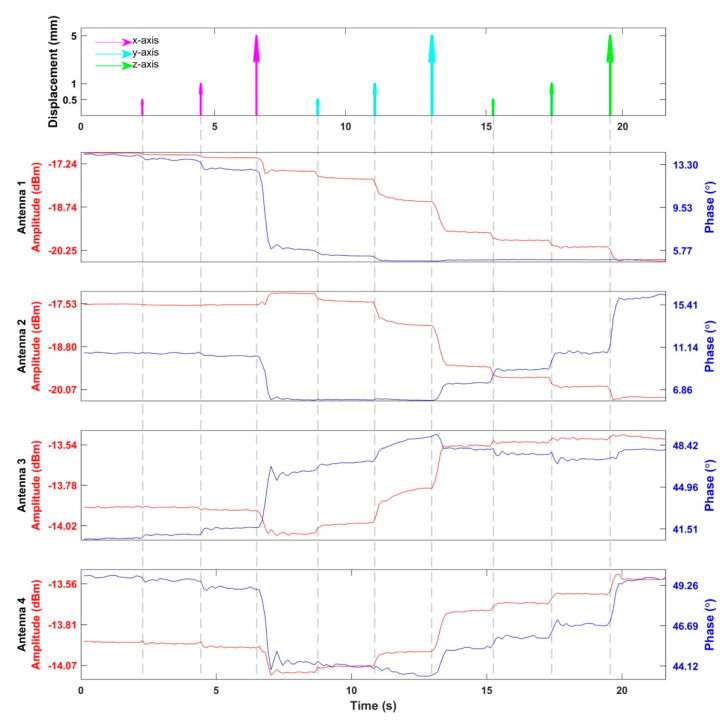
Detection of head motion. The top figure indicates the timing and displacements of the movements of the head model. There were three movements with the distance of 0.5, 1.0, and 5.0 mm along each direction. The following four figures show the amplitude and phase of the reflected RF signals from the SGBT antennas detected by the supervision module. The curves drawn in red represent the amplitudes of the reflected signals, while the curves in blue represent the phases of the reflected signal relative to the transmitted/reference signal.

Following the conclusions drawn from the EMF simulations of the human head models, the RF supervision module’s applicability for motion detection was examined in experiments using a 3D printed human head model as an OUI. The results of the head motion detection experiment are summarized in Figure 9. Three displacements with different magnitudes (0.5, 1.0, and 5.0 mm) were repeated on the X-, Y-, and Z-axes. All the movements caused signal changes which scaled with the displacements. Larger signal changes corresponded to larger displacements. Movements along the X-axis were detected by antennas 1, 3, and 4, including displacements as small as 0.5 mm. Movements along the Y- and Z-axes were captured by all antennas including shifts with a 0.5 mm magnitude.

### 3.4. Analysis of Impact of Power and Phase Errors on Hyperthermia Performance

**Figure 10:**
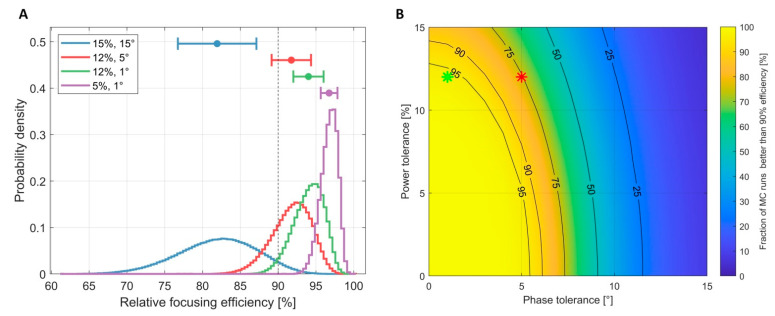
(**A**) Probability distribution of the focusing efficiency relative to the optimal RF power and phase setting. The four curves represent four Monte-Carlo simulations with different error ranges (blue: 15% error in power and 15° in phase; red: 12% error in power and 5° in phase, representing the performance of the 7.0 T Siemens MR scanner used in Section 2.3; green: 12% error in power and 1° in phase, representing the characteristics of the proposed supervision module; purple: 5% error in power and 1° in phase). (**B**) Percentage of Monte-Carlo runs that yielded a performance ≥ 90% depending on power and phase tolerances. The red star indicates the performance of the 7.0 T Siemens MR scanner while the green star indicates the performance of the proposed RF supervision module. This graph demonstrates that phase errors, especially, quickly led to a loss of excitation fidelity.

Figure 10 depicts results from the Monte-Carlo (MC) analyses of the impact of the power and phase errors of RF signals on hyperthermia performance. The performance of the proposed RF supervision module, which had a power tolerance of 12% and a phase tolerance of 1°, was compared with that of the RF supervision system of a multi-channel parallel transmission (pTX) system of a state-of-the-art 7.0 T MRI scanner (Magnetom, Siemens Healthineers, Erlangen, Germany) that had a power tolerance of 12% and a phase tolerance of 5° [44]. The proposed RF supervision module outperformed the RF supervision system of the commercial pTX system, as outlined in Figure 10A for the relative focusing efficiency. Our Monte-Carlo simulations demonstrated that phase errors, especially, heavily contributed to a loss of excitation and HT fidelity (Figure 10B).

## 4. Discussion

### 4.1. System Characterization

The final power level readings of the RF input channels depended on the power level of the reference channel. During calibration, the reference input of the meter chip was set to –30 dBm due to the fact that the largest linearity and detection range were achieved at this point. In field operation, the signal conditioning circuit was activated to adjust the reference signal to this point (−30 dBm). Hence, the power level of the reference channel was omitted in Equation (2). Due to the adoption of the 16-bit analog-to-digital converter, a theoretical resolution of 2.6 × 10^−3^ dBm was obtained. The attenuators included in the signal conditioning circuits further extend the linear detection range to 80 dB (from −50 to 30 dBm). It is notable that the signal frequency had a great impact on the behavior of the power meters. A maximum variation of 5.72 dB (3.73 times in power difference) was found for the same output from the meter when the frequency changed. Many of the existing power meter implementations for RF hyperthermia and/or for SAR monitoring in MRI employ similar logarithmic power detectors [22,23,45,46,47]. These setups mostly operate at a single frequency or a narrow band of frequencies. They typically utilize a lookup table to translate the meter outputs to power levels, where the impact of frequency is not accounted for. For example, the HYPERcollar [47] system, which utilizes the same power detector chip used in our work, operates at 433.92 MHz. However, recent advancements in hyperthermia, including numerical simulations [24,25] and novel algorithms [26,27,28,29], have suggested the use of a broad range of frequencies to enhance HT delivery. To turn these insights into clinical applications and value, it is essential to characterize and compensate for the impact of the signal frequency on the power meters for ensuring the safety and efficiency of HT treatments. 

The phase meter demonstrated a great linearity in the range of 5°–175°, regardless of the signal frequency. A theoretical resolution of 7.2 × 10^−3^ (°) was obtained. The phase meter was ambiguous for phases that were symmetrical around 180°, e.g., phase = 90° and phase = 270° resulted in the same ADC reading in the curves. Considering the phase meter measures for the phase difference between the RF input channel and the reference channel, this ambiguity could be eliminated by observing the result from shifting the phase of the reference signal by a small amount of phase angles. In practice, this approach was conveniently implemented by using the phase shifting function of the RF signal generator [15] developed in our group. Thus, for monitoring/regulating one RF channel that contains forward and reflected signals, only two power and phase detectors (AD8302) need to be used. In this way, the proposed design can reduce system cost and complexity compared with similar implementation [45] that uses three power and phase detectors for one RF channel.

The maximum sampling rate of the proposed supervision module was found to be 28.4 samples per second, with each sample containing the power and phase information of all the channels. The quality assurance guidelines of the European Society of Hyperthermic Oncology (ESHO) for hyperthermia [48,49,50] require a computerized data acquisition system to record all system control parameters including signal frequency, phase, power level (forward and reflected), and any changes during treatment in these settings. According to the ESHO guidelines, the sampling interval for the power level should be less than 20 s [48]. The sampling rate of the proposed RF supervision module meets these requirements, and it is found to be higher than the sampling rate of the commonly used BSD-2000 3D hyperthermia system that updates the power level every two seconds [51]. The high sampling rate provided by our RF supervision module affords the use of a larger number of RF channels with one supervision module by multiplexing the input signals.

### 4.2. Impact of Power and Phase Errors on Hyperthermia Performance

The Monte-Carlo analyses revealed that the performance of an RF-based hyperthermia system is more sensitive to the phase errors of RF signals. The proposed supervision module outperformed the supervision system of a state-of-the-art 7.0 T MR scanner’s multi-channel parallel transmission system. For the clinical application of an HT system, an error analysis taking all tolerances of the integrated RF system into account is indispensable to adjust the prescribed treatment power level. Hence, the performance of a hyperthermia system is limited by its weakest link—an RF power amplifier with a supposedly perfect power and phase response is of limited use if its operation cannot be monitored by a supervision module with similar accuracy and precision. The Monte-Carlo simulations showed that a tight control on measurement errors translates into a higher fidelity reproduction of the planned RF field superposition. Phase errors, especially, quickly lead to a degradation of the focusing capabilities.

### 4.3. RFPA Characterization and RF Heating Experiments

Though the two RFPAs used in this work shared the same design and implementation, RFPA characterization provided different characteristics. The maximum variation of the gain along frequency for an RFPA could be as large as 8.3 dB (6.76 times). The average difference of the gain between the two RFPAs was 5.5 dB (3.55 times) with a varying frequency. The performance variations of the RFPAs were related to factors such as variations of electronic components and temperature. These variations measured with the supervision module are not unique to our home-built amplifiers. Previous studies on quality assurance in hyperthermia performed on different types of systems have reported similar outcomes [52,53,54,55,56]. These studies employed vector voltmeters to validate hyperthermia systems. However, vector voltmeters are already obsolete. 

The RF heating experiments underlined the need of a closed-loop control system. MR thermometry revealed a supervised heating pattern that was in accordance with the reference obtained from the numerical simulation. The temperature maps obtained for unsupervised heating were distorted compared to the reference. The peak of the interference pattern in the middle of the phantom was shifted to channel 2 for 16 mm, which translated into a phase difference between the two channels of approximately 80°. This was in accordance with the results derived from our RFPA characterization experiments. By employing the RF supervision module in the control loop, component- and temperature-dependent deviations and uncertainties covered by the control loop could be eliminated.

In multi-channel phased array systems, the power amplifier is not the only source of error. The phase-amplitude control can be substantially affected by factors such as the coupling and mismatches of RF channels [57,58]. Raskmark et al. proposed an algorithm that uses the scattering matrix (S-matrix) to describe multi-channel RF systems and correct system errors [56,59]. More recently, the self-calibration technique was introduced into hyperthermia antenna arrays to compensate for disturbances and patient mismatches in real-time [30]. The self-calibration algorithms rely on the S-matrix measurements of an antenna array. Combined with the multi-channel signal generator [15] and bi-directional couplers, the proposed supervision module is capable of measuring the S-matrix of an N-port array applicator. Hence, the aforementioned algorithms can be implemented without costly, multi-port vector network analyzers. This advancement helps to reduce the costs of HT systems and increases the safety and efficiency of hyperthermia treatments.

### 4.4. Impact of Head Motion

Patient positioning is an important factor to be considered for high-quality HT delivery and patient safety. The ESHO guidelines [49] suggest an accuracy of patient positioning of 1 cm. A previous study [60] using a BSD-2000 Sigma 60 system operating in the frequency range of 75–140 MHz showed that precise positioning is mandatory for high-quality hyperthermia treatments and demonstrated that the positioning error should be less than 1 cm. A sensitivity study [61] on optimal temperature distribution with respect to patient positioning indicated that the sensitivity increases steeply with the operating frequency. Since higher frequencies and a larger number of independent RF antennas enhance HT quality [24,32,62], new RF applicators tailored for brain HT accommodate broad band antennas and high-density arrangements [11,14,63,64]. Patient displacements due to motion in these applicators are inevitable, even for compliant subjects considering the long treatment time (60–90 min). Head motion could be caused by multiple sources including discomfort or pain from the treatment, breathing, coughing, and swallowing. Following the ESHO guidelines, we simulated the impact of a 5 mm displacement to SAR distribution using a synthetic tumor head model and a clinically relevant head model. The SAR distributions of the displaced position were inferior to that of the center position for both head models. Our results indicate that RF exposure in the small, synthetic target volume might not have significantly suffered from malpositioning. However, the higher RF exposure of the healthy tissue due to the offset between the head position used for HTP and the real scenario could cause patient discomfort and even local damage to healthy tissue. While the thermal treatment of other tumor sites often relies on patient feedback [65], it is important to consider the lack of pain sensory neurons (nociceptors) in the brain [66]. This underlines the need of a high level of monitoring and control over patient position. In the clinically relevant tumor model, the imbalance caused by the 5 mm shift in patient positioning was found to be even more pronounced. The drop observed in therapeutic metrics was as high as 22.3% for the local SAR_10g,max_(tumor) and 33.45% in target coverage. While the average exposure of the healthy tissue also dropped by over 10%, a local maximum increased by the same amount. This indicates that for such a treatment plan, the treatment efficacy would collapse in the case of a patient displacement of only 5 mm, again highlighting the importance of controlling and monitoring the head position prior to and during thermal intervention.

Our RF supervision module addresses this challenge and facilitates the detection of subtle head displacements. The use of a water bolus increased the electrical coupling between the antenna and the patient and enabled the detection of submillimeter displacements. By monitoring the reflected signal from the antennas, the RF supervision module provides a cost-effective solution to patient position monitoring. This is particularly relevant for hyperthermia treatment planning because the position of the patient model must accurately resemble the position during the clinical application to achieve optimal SAR/temperature steering [32,33]. Patient displacement from the optimized setting leads to a decreased efficiency and constitutes a potential safety hazard. As algorithms advance and the available computational power grows, on-line adaptive HTP becomes more viable [67,68,69,70,71]. Patient motion information provided by the multi-channel RF supervision module can be an important input to the HTP system to trigger the re-optimization of power and phase settings in real-time for the benefit of safe and high quality HT delivery. 

## 5. Conclusions

To achieve precise, efficient, and safe RF-induced hyperthermia treatment at a broad range of frequencies, the RF signals connected to the antennas need to be supervised in real-time. In addition, it is also very important to monitor and control the patient position during thermal interventions, especially for the brain region. This work demonstrates the development, implementation, evaluation, validation, and application of a multi-channel RF supervision module that meets the technical requirements in hyperthermia and provides a cost-effective solution for broad-band RF signal supervision and patient monitoring. The proposed supervision module is a key component for a hyperthermia hardware system, could provide important data for a hyperthermia treatment planning system, and forms a technological basis for future ThermalMR applications.

## Figures and Tables

**Table 1:** Metrics used for the evaluation of the impact of motion on the RF heating of the Duke head model (Duke) and the CRM of the head. The Duke 1 and CRM 1 columns show the numerical results of simulations (Figure 8A,C) with the head models being placed in the center of the RF applicator. The Duke 2 and CRM 2 columns are the numerical results of simulations (Figure 8B,D) with the human head models being placed off-center (5 mm displacement upwards). SAF: SAR amplification factor; THQ: tumor-to-hotspot quotient; TC: target coverage.

Metrics	Duke 1 (A)	Duke 2 (B)	Relative Difference (%)	CRM 1 (C)	CRM 2 (D)	Relative Difference (%)
SAR_10g,max_(tumor), W/kg	64.56	61.47	−4.79	84.50	65.65	−22.31
SAR_10g,max_(healthy), W/kg	40	44.06	+10.15	40	44.44	+11.1
SAR_10g,mean_(tumor), W/kg	48.06	45.92	−4.45	42.77	36.34	−15.03
SAR_10g,mean_(healthy), W/kg	18.36	18.51	+0.82	19.46	16.95	−12.90
P_tumor_/V_tumor_, W/L	61.19	58.51	−4.38	36.56	30.92	−15.43
SAF	2.62	2.48	−5.34	2.20	2.14	−2.73
THQ	1.23	1.15	−9.84	1.10	1.01	−8.18
TC(SAR_tumor_ > SAR_Lim_)	85.9%	78.8%	−8.27	57.1%	38%	−33.45

## Data Availability

Not applicable.

## References

[1] Ostrom Q.T., Cioffi G., Gittleman H., Patil N., Waite K., Kruchko C., Barnholtz-Sloan J.S. (2019). CBTRUS Statistical Report: Primary Brain and Other Central Nervous System Tumors Diagnosed in the United States in 2012–2016. Neuro. Oncol..

[2] Tan A.C., Ashley D.M., López G.Y., Malinzak M., Friedman H.S., Khasraw M. (2020). Management of glioblastoma: State of the art and future directions. CA Cancer J. Clin..

[3] De Vleeschouwer S. (2017). Glioblastoma.

[4] Lee Titsworth W., Murad G.J.A., Hoh B.L., Rahman M. (2014). Fighting fire with fire: The revival of thermotherapy for gliomas. Anticancer Res..

[5] Overgaard J., Bentzen S.M., Gonzalez Gonzalez D., Hulshof M., Arcangeli G., Dahl O., Mella O. (1995). Randomised trial of hyperthermia as adjuvant to radiotherapy for recurrent or metastatic malignant melanoma. Lancet.

[6] Wust P., Hildebrandt B., Sreenivasa G., Rau B., Gellermann J., Riess H., Felix R., Schlag P.M. (2002). Hyperthermia in combined treatment of cancer. Lancet Oncol..

[7] Cihoric N., Tsikkinis A., van Rhoon G., Crezee H., Aebersold D.M., Bodis S., Beck M., Nadobny J., Budach V., Wust P. (2015). Hyperthermia-related clinical trials on cancer treatment within the ClinicalTrials.gov registry. Int. J. Hyperth..

[8] Van der Zee J., González D., van Rhoon G.C., van Dijk J.D.P., van Putten W.L.J., Hart A.A. (2000). Comparison of radiotherapy alone with radiotherapy plus hyperthermia in locally advanced pelvic tumours: A prospective, randomised, multicentre trial. Lancet.

[9] Franckena M., Stalpers L.J.A., Koper P.C.M., Wiggenraad R.G.J., Hoogenraad W.J., van Dijk J.D.P., Wárlám-Rodenhuis C.C., Jobsen J.J., van Rhoon G.C., van der Zee J. (2008). Long-term improvement in treatment outcome after radiotherapy and hyperthermia in locoregionally advanced cervix cancer: An update of the Dutch Deep Hyperthermia Trial. Int. J. Radiat. Oncol. Biol. Phys..

[10] Roussakow S.V. (2017). Clinical and economic evaluation of modulated electrohyperthermia concurrent to dose-dense temozolomide 21/28 days regimen in the treatment of recurrent glioblastoma: A retrospective analysis of a two-centre German cohort trial with systematic comparison and effect-to-treatment analysis. BMJ Open.

[11] Oberacker E., Kuehne A., Oezerdem C., Nadobny J., Weihrauch M., Beck M., Zschaeck S., Diesch C., Eigentler T.W., Waiczies H. (2020). Radiofrequency applicator concepts for thermal magnetic resonance of brain tumors at 297 MHz (7.0 Tesla). Int. J. Hyperth..

[12] Niendorf T., Oezerdem C., Ji Y., Oberacker E., Kuehne A., Waiczies H., Winter L. Radiative RF antenna arrays for cardiac, brain and thermal magnetic resonance at ultrahigh and extreme magnetic field strengths: Concepts, electromagnetic field simulations and applications. Proceedings of the 2017 International Conference on Electromagnetics in Advanced Applications (ICEAA).

[13] Ji Y., Hoffmann W., Pham M., Dunn A.E., Han H., Özerdem C., Waiczies H., Rohloff M., Endemann B., Boyer C. (2018). High peak and high average radiofrequency power transmit/receive switch for thermal magnetic resonance. Magn. Reson. Med..

[14] Eigentler T.W., Winter L., Han H., Oberacker E., Kuehne A., Waiczies H., Schmitter S., Boehmert L., Prinz C., Trefna H.D. (2020). Wideband Self-Grounded Bow-Tie Antenna for Thermal MR. NMR Biomed..

[15] Han H., Eigentler T.W., Wang S., Kretov E., Winter L., Hoffmann W., Grass E., Niendorf T. (2020). Design, Implementation, Evaluation and Application of a 32-Channel Radio Frequency Signal Generator for Thermal Magnetic Resonance Based Anti-Cancer Treatment. Cancers.

[16] Young I.R., Hand J.W., Oatridge A., Prior M.V. (1994). Modeling and observation of temperature changes in vivo using MRI. Magn. Reson. Med..

[17] Winter L., Oberacker E., Paul K., Ji Y., Oezerdem C., Ghadjar P., Thieme A., Budach V., Wust P., Niendorf T. (2016). Magnetic resonance thermometry: Methodology, pitfalls and practical solutions. Int. J. Hyperth..

[18] De Denis Senneville B., Quesson B., Moonen C.T.W. (2005). Magnetic resonance temperature imaging. Int. J. Hyperth..

[19] Paulides M.M., Curto S., Wu M., Winter L., van Rhoon G.C., Yeo D.T.B. (2017). Advances in magnetic resonance guided radiofrequency hyperthermia. Proceedings of the 11th European Conference on Antennas and Propagation (EUCAP), Paris, France, 19–24 March 2017.

[20] Brote I., Orzad S., Kraff O., Maderwald S., Quick H.H., Yazdanbakhsh P., Solbach K., Wicklow K., Bahr A., Bolz T. A Multi-Channel SAR Prediction and Online Monitoring System at 7T. Proceedings of the 17th Annual Meeting of ISMRM.

[21] Bakker J., Paulides M., van Rhoon G., Schippers H., Ter Meer T. Development of a gain & phase detector for a head & neck electromagnetic hyperthermia applicator. Proceedings of the Joint 9th International Conference on Electromagnetics in Advanced Applications, ICEAA 2005 and 11th European Electromagnetic Structures Conference, EESC 2005.

[22] Yan X., Shi L., Feng B., Wang Z., Wei S., Ma C., Wei L., Xue R. A Multi-Channel Real-Time Power Monitoring System for SAR Estimation Using FPGA in High Field MRI. Proceedings of the 24th Annual Meeting of ISMRM.

[23] Kudielka G., Vogel M., Loew W. Realisation of a flexible power measurement application for Parallel Transmit. Proceedings of the 17th Annual Meeting of the ISMRM.

[24] Guérin B., Villena J.F., Polimeridis A.G., Adalsteinsson E., Daniel L., White J.K., Rosen B.R., Wald L.L. (2018). Computation of ultimate SAR amplification factors for radiofrequency hyperthermia in non-uniform body models: Impact of frequency and tumour location. Int. J. Hyperth..

[25] Takook P., Dobsicek Trefna H., Zeng X., Fhager A., Persson M. (2017). A Computational Study Using Time Reversal Focusing for Hyperthermia Treatment Planning. PIER B.

[26] Bellizzi G.G., Crocco L., Battaglia G.M., Isernia T. (2017). Multi-Frequency Constrained SAR Focusing for Patient Specific Hyperthermia Treatment. IEEE J. Electromagn. RF Microw. Med. Biol..

[27] Kok H.P., van Stam G., Bel A., Crezee J. (2015). A mixed frequency approach to optimize locoregional RF hyperthermia. Proceedings of the 2015 European Microwave Conference (EuMC 2015), Paris, France, 7–10 September 2015.

[28] Zastrow E., Hagness S.C., van Veen B.D., Medow J.E. (2011). Time-multiplexed beamforming for noninvasive microwave hyperthermia treatment. IEEE Trans. Biomed. Eng..

[29] Kuehne A., Oberacker E., Waiczies H., Niendorf T. (2020). Solving the Time- and Frequency-Multiplexed Problem of Constrained Radiofrequency Induced Hyperthermia. Cancers.

[30] Zanoli M., Persson M., Trefna H.D. (2018). Self-calibration algorithms for microwave hyperthermia antenna arrays. Proceedings of the EuCAP 2018 12th European Conference on Antennas and Propagation (EuCAP 2018), London, UK, 9–13 April 2018.

[31] Wust P., Weihrauch M. (2009). Hyperthermia classic commentary: ‘Simulation studies promote technological development of radiofrequency phased array hyperthermia’ by Peter Wust et al., International Journal of Hyperthermia 1996;12:477-494. Int. J. Hyperth..

[32] Kok H.P., Wust P., Stauffer P.R., Bardati F., van Rhoon G.C., Crezee J. (2015). Current state of the art of regional hyperthermia treatment planning: A review. Radiat. Oncol..

[33] De Greef M., Kok H.P., Bel A., Crezee J. (2011). 3D versus 2D steering in patient anatomies: A comparison using hyperthermia treatment planning. Int. J. Hyperth..

[34] Buikman D., Helzel T., Röschmann P. (1988). The rf coil as a sensitive motion detector for magnetic resonance imaging. Magn. Reson. Imaging.

[35] Hess A.T., Tunnicliffe E.M., Rodgers C.T., Robson M.D. (2018). Diaphragm position can be accurately estimated from the scattering of a parallel transmit RF coil at 7 T. Magn. Reson. Med..

[36] De Poorter J., de Wagter C., de Deene Y., Thomsen C., Ståhlberg F., Achten E. (1995). Noninvasive MRI thermometry with the proton resonance frequency (PRF) method: In vivo results in human muscle. Magn. Reson. Med..

[37] Ishihara Y., Calderon A., Watanabe H., Okamoto K., Suzuki Y., Kuroda K. (1995). A precise and fast temperature mapping using water proton chemical shift. Magn. Reson. Med..

[38] Kuroda K., Oshio K., Chung A.H., Hynynen K., Jolesz F.A. (1997). Temperature mapping using the water proton chemical shift: A chemical shift selective phase mapping method. Magn. Reson. Med..

[39] Restivo M.C., van den Berg C.A.T., van Lier A.L.H.M.W., Polders D.L., Raaijmakers A.J.E., Luijten P.R., Hoogduin H. (2016). Local specific absorption rate in brain tumors at 7 tesla. Magn. Reson. Med..

[40] 40. Nadobny J., Weihrauch M., Zschaeck S., Lim A., Beck M., Chrzon B.C. (2018). Fast and efficient generation of patient models for hyperthermia based on radiation therapy contours. Proceedings of the 32nd Annual Meeting of the European Society for Hyperthermic Oncology.

[41] (2003). IEEE Recommended Practice for Determining the Peak Spatial-Average Specific Absorption Rate (SAR) in the Human Head from Wireless Communications Devices: Measurement Techniques.

[42] Han H., Moritz R., Oberacker E., Waiczies H., Niendorf T., Winter L. (2017). Open Source 3D Multipurpose Measurement System with Submillimetre Fidelity and First Application in Magnetic Resonance. Sci. Rep..

[43] Canters R.A.M., Wust P., Bakker J.F., van Rhoon G.C. (2009). A literature survey on indicators for characterisation and optimisation of SAR distributions in deep hyperthermia, a plea for standardisation. Int. J. Hyperth..

[44] (2012). Siemens Magnetom 7T Tx Array Systems Operator Manual.

[45] Bakker J.F., Paulides M.M., Westra A.H., Schippers H., van Rhoon G.C. (2010). Design and test of a 434 MHz multi-channel amplifier system for targeted hyperthermia applicators. Int. J. Hyperth..

[46] Orzada S., Solbach K., Gratz M., Brunheim S., Fiedler T.M., Johst S., Bitz A.K., Shooshtary S., Abuelhaija A., Voelker M.N. (2019). A 32-channel parallel transmit system add-on for 7T MRI. PLoS ONE.

[47] Paulides M.M., Bakker J.F., Neufeld E., van der Zee J., Jansen P.P., Levendag P.C., van Rhoon G.C. (2007). Winner of the “New Investigator Award” at the European Society of Hyperthermia Oncology Meeting 2007. The HYPERcollar: A novel applicator for hyperthermia in the head and neck. Int. J. Hyperth..

[48] Hand J.W., Lagendijk J.J., Bach Andersen J., Bolomey J.C. (1989). Quality assurance guidelines for ESHO protocols. Int. J. Hyperth..

[49] Lagendijk J.J., van Rhoon G.C., Hornsleth S.N., Wust P., de Leeuw A.C., Schneider C.J., van Dijk J.D., van der Zee J., van Heek-Romanowski R., Rahman S.A. (1998). ESHO quality assurance guidelines for regional hyperthermia. Int. J. Hyperth..

[50] Bruggmoser G., Bauchowitz S., Canters R., Crezee H., Ehmann M., Gellermann J., Lamprecht U., Lomax N., Messmer M.B., Ott O. (2012). Guideline for the clinical application, documentation and analysis of clinical studies for regional deep hyperthermia: Quality management in regional deep hyperthermia. Strahlenther. Onkol..

[51] Pyrexar Medical BSD-2000-3D. https://www.pyrexar.com/hyperthermia/bsd-2000-3d.

[52] Lee W.M., Ameziane A., van den Biggelaar A.M.C., Rietveld P.J.M., van Rhoon G.C. (2003). Stability and accuracy of power and phase measurements of a VVM system designed for online quality control of the BSD-2000 (-3D) DHT system. Int. J. Hyperth..

[53] Kongsli J., Hjertaker B.T., Frøystein T. (2006). Evaluation of power and phase accuracy of the BSD Dodek amplifier for regional hyperthermia using an external vector voltmeter measurement system. Int. J. Hyperth..

[54] Lamprecht U., Gromoll C., Hehr T., Buchgeister M., Bamberg M. (2000). An on-line phase measurement system for quality assurance of the BSD 2000. Part II: Results of the phase measurement system. Int. J. Hyperth..

[55] Straube W.L., Moros E.G., Myerson R.J. (1995). Phase stability of a clinical phased array system for deep regional hyperthermia. Int. J. Hyperth..

[56] Hornsleth S.N., Frydendal L., Mella O., Dahl O., Raskmark P. (1997). Quality assurance for radiofrequency regional hyperthermia. Int. J. Hyperth..

[57] Wust P., Beck R., Berger J., Fähling H., Seebass M., Wlodarczyk W., Hoffmann W., Nadobny J. (2000). Electric field distributions in a phased-array applicator with 12 channels: Measurements and numerical simulations. Med. Phys..

[58] Wust P., Fähling H., Wlodarczyk W., Seebass M., Gellermann J., Deuflhard P., Nadobny J. (2001). Antenna arrays in the SIGMA-eye applicator: Interactions and transforming networks. Med. Phys..

[59] Raskmark P., Larsen T., Hornsleth S.N. (1994). Multi-applicator hyperthermia system description using scattering parameters. Int. J. Hyperth..

[60] Canters R.A.M., Franckena M., Paulides M.M., van Rhoon G.C. (2009). Patient positioning in deep hyperthermia: Influences of inaccuracies, signal correction possibilities and optimization potential. Phys. Med. Biol..

[61] Seebass M., Beck R., Gellermann J., Nadobny J., Wust P. (2001). Electromagnetic phased arrays for regional hyperthermia: Optimal frequency and antenna arrangement. Int. J. Hyperth..

[62] Paulides M.M., Vossen S.H.J.A., Zwamborn A.P.M., van Rhoon G.C. (2005). Theoretical investigation into the feasibility to deposit RF energy centrally in the head-and-neck region. Int. J. Radiat. Oncol. Biol. Phys..

[63] Schooneveldt G., Dobšíček Trefná H., Persson M., de Reijke T.M., Blomgren K., Kok H.P., Crezee H. (2019). Hyperthermia Treatment Planning Including Convective Flow in Cerebrospinal Fluid for Brain Tumour Hyperthermia Treatment Using a Novel Dedicated Paediatric Brain Applicator. Cancers.

[64] Turner P.F., Ellsworth J. (2020). Apparatus and Method for Creating Small Focus Deep Hyperthermia in Tissues of the Brain. U.S. Patent.

[65] Van der Wal E., Franckena M., Wielheesen D.H.M., van der Zee J., van Rhoon G.C. (2008). Steering in locoregional deep hyperthermia: Evaluation of common practice with 3D-planning. Int. J. Hyperth..

[66] Dubin A.E., Patapoutian A. (2010). Nociceptors: The sensors of the pain pathway. J. Clin. Investig..

[67] Kok H.P., Ciampa S., de Kroon-Oldenhof R., Steggerda-Carvalho E.J., van Stam G., zum Vörde Sive Vörding P.J., Stalpers L.J.A., Geijsen E.D., Bardati F., Bel A. (2014). Toward online adaptive hyperthermia treatment planning: Correlation between measured and simulated specific absorption rate changes caused by phase steering in patients. Int. J. Radiat. Oncol. Biol. Phys..

[68] Cheng K.-S., Stakhursky V., Stauffer P., Dewhirst M., Das S.K. (2007). Online feedback focusing algorithm for hyperthermia cancer treatment. Int. J. Hyperth..

[69] Ranneberg M., Weiser M., Weihrauch M., Budach V., Gellermann J., Wust P. (2010). Regularized antenna profile adaptation in online hyperthermia treatment. Med. Phys..

[70] Stakhursky V.L., Arabe O., Cheng K.-S., Macfall J., Maccarini P., Craciunescu O., Dewhirst M., Stauffer P., Das S.K. (2009). Real-time MRI-guided hyperthermia treatment using a fast adaptive algorithm. Phys. Med. Biol..

[71] Weihrauch M., Wust P., Weiser M., Nadobny J., Eisenhardt S., Budach V., Gellermann J. (2007). Adaptation of antenna profiles for control of MR guided hyperthermia (HT) in a hybrid MR-HT system. Med. Phys..

